# Five year trajectories of the mental and physical SF‐36 summary scores in men: results from the PREADViSE trial

**DOI:** 10.1002/alz.084871

**Published:** 2025-01-09

**Authors:** Xiuhua Ding, Richard Charnigo, Frederick A Schmitt, Richard J Kryscio, Erin L. Abner

**Affiliations:** ^1^ Western Kentucky University, Bowling Green, KY USA; ^2^ University of Kentucky, Lexington, KY USA; ^3^ University of Kentucky College of Medicine, Lexington, KY USA; ^4^ Sanders‐Brown Center on Aging, Lexington, KY USA; ^5^ College of Public Health, University of Kentucky, Lexington, KY USA; ^6^ Sanders‐Brown Center on Aging, University of Kentucky, Lexington, KY USA

## Abstract

**Background:**

Previous studies have shown that SF36 summary scores predict incident dementia. However, it is unclear whether SF36 summary scores follow different temporal trajectories. Thus, the goal of this study is to examine possible trajectories for SF36 summary scores.

**Method:**

A subset of 2,746 Prevention of Alzheimer’s Disease (AD) by Vitamin E and Selenium (PREADViSE) participants who completed SF‐36 assessments at annual visits was included in the current analysis. First, group‐based trajectory modeling (GBTM) was applied to fit SF‐36 Mental Component summary (MCS) scores and Physical Component Summary (PCS) scores. The final models were selected based on perceptions of interpretability and reproducibility with the assistance of the Bayesian information criterion (BIC). Then joint trajectory modeling was used to examine overlaps between trajectory groups based on MCS scores and trajectory groups based on PCS scores.

**Result:**

Four trajectories were identified for each of MCS and PCS, when examined separately. In terms of shape, MCS trajectories were described as stable (∼81.3% of subjects), curvilinear decline (∼ 4.3%), linear decline (∼ 11.2%) and slight increase (∼ 3.2%), while PCS trajectories were described as stable (∼48.7%) and slight decline (∼51.3% encompassing three groups). Joint trajectory modelling showed overlaps suggesting that MCS and PCS trajectories are not independent.

**Conclusion:**

Both MCS scores and PCS scores showed multiple trajectories over time; some were stable, and some exhibited decline. We were also able to estimate how many subjects belonged to each group and to quantify overlaps in group membership for MCS and PCS scores.

